# Misregulated alternative splicing in endometriosis: a role for aberrant mRNA variants in endometriotic cell growth

**DOI:** 10.1038/s41420-026-03015-z

**Published:** 2026-03-15

**Authors:** Venkata Naga Goutham Davuluri, Michelle Dias, Roxanna Llinas, Neha Kamath, Pooja Popli, Shannon M. Hawkins, Chandrakant Tayade, Elise T. Courtois, Hari Krishna Yalamanchili, Ramakrishna Kommagani

**Affiliations:** 1 Department of Pathology and Immunology, Baylor College of Medicine, Houston, TX USA; 2 Department of Pediatrics, Baylor College of Medicine, Houston, TX USA; 3 Jan and Dan Duncan Neurological Research Institute, Texas Children’s Hospital, Houston, TX 77030, USA; USDA/ARS Children’s Nutrition Research Center, Department of Pediatrics, Baylor College of Medicine, Houston, TX USA; 4 Department of Obstetrics and Gynecology, Indiana University School of Medicine, Indianapolis, IN USA; 5 Department of Biomedical and Molecular Sciences, Queen’s University, Kingston, ON Canada; 6 The Jackson Laboratory for Genomic Medicine, Farmington, CT USA; 7 UConn Health School of Medicine, OB/Gyn Department, Farmington, CT USA; 8 USDA/ARS Children’s Nutrition Research Center, Department of Pediatrics, Baylor College of Medicine, Houston, TX USA; 9 Department of Molecular Virology and Microbiology, Baylor College of Medicine, Houston, TX USA; 10 Center for Drug Discovery, Baylor College of Medicine, Houston, USA

**Keywords:** Alternative splicing, Endocrine reproductive disorders

## Abstract

Endometriosis is a chronic gynecological disorder marked by the growth of endometrial-like tissue outside the uterus, often leading to pelvic pain, inflammation, and infertility. Despite its global prevalence, diagnosis remains delayed, and effective non-surgical treatments are lacking. While recent transcriptomic studies have identified mRNA transcript changes in ectopic lesions, the contribution of alternative pre-mRNA splicing, a key posttranscriptional regulatory layer, remains largely unknown. In this study, we performed a comprehensive analysis of alternative splicing (AS) events in endometriotic lesions using transcriptomic data. We uncovered distinct alterations in alternative splicing events are associated with endometriosis, highlighting a previously underappreciated layer of gene regulation. Specifically, we discovered that AS events, including exon skipping (SE) and intron retention (IR), were more prevalent than other events, and altered AS events correlated with transcriptomic variation in lesions. We identified two genes, *GALNT7* and *ZNF28*, with significantly reduced exon inclusion in epithelial cells of lesions, potentially resulting in decreased levels of mature transcripts. Functional assays showed that knockdown of *GALNT7* and *ZNF28*, or of critical exons within these genes, increased cellular proliferation, supporting their potential roles as growth‑suppressive genes in endometriotic cells. Together, this study broadens our understanding of transcriptomic dysregulation and highlights misregulated alternative splicing as a potential contributor to endometriotic cell growth and disease progression.

## Introduction

Endometriosis is a chronic condition, characterized by the presence and growth of endometrial-like tissues or “lesions”, outside the uterus [1]. Affecting nearly 10% of reproductive-aged women worldwide, this disease impacts an estimated 190 million individuals globally and is a major cause of infertility and pelvic pain [2]. Lesions frequently form on the ovaries, fallopian tubes, peritoneum, and other pelvic organs, and in some cases, can invade deeper muscular structures. These ectopic tissues histologically resemble the uterine endometrium and respond to hormonal signals, triggering inflammation, fibrosis, and chronic pain. Clinically, endometriosis presents with a wide range of symptoms, including dysmenorrhea (painful menstruation), dyspareunia (painful intercourse), chronic pelvic pain, gastrointestinal disturbances, and infertility [3]. However, despite its high prevalence and serious quality-of-life impacts, endometriosis remains poorly understood and is frequently underdiagnosed, with an average delay of seven years from symptom onset to diagnosis [3, 4]. This delay is further compounded by the disease’s heterogeneity and a lack of comprehensive epidemiological and clinical data, which together obscure its full biological and clinical scope. Current treatments, including pain medications, hormonal modulators and surgical excision of lesions and total hysterectomies, offer symptomatic relief but are limited by recurrence rates and side effects, underscoring the need to better understand the underlying pathophysiological mechanisms [2]. The pathogenesis of endometriosis is believed to involve retrograde menstruation, where endometrial cells are refluxed through the fallopian tubes into the peritoneal cavity. However, retrograde menstruation occurs in most women, and only a subset goes on to develop endometriosis, suggesting that additional factors such as immune dysfunction, environmental toxins, hormonal imbalances, and cellular reprogramming contribute to lesion establishment and progression [5–7]. Given its widespread impact and chronic and recurring nature, the development of robust and preventive strategies is crucial for improving the lives of millions of women worldwide.

At the molecular level, endometriosis is influenced by genetic, environmental, hormonal, and immune factors. Genome-wide association studies (GWAS) have identified multiple single-nucleotide polymorphisms (SNPs) associated with increased endometriosis risk. Many SNPs identified are in noncoding regions, implicating regulatory elements in disease susceptibility [8]. Epigenetic changes, including altered DNA methylation and histone modifications, have also been observed in endometriotic lesions and are thought to contribute to aberrant gene expression patterns. More recently, transcriptomic profiling approaches such as RNA-sequencing (RNA-seq) and single-cell RNA-sequencing (scRNA-seq) have proven valuable in providing critical insights into the transcriptional landscapes of endometriotic tissues. These analyses have revealed widespread differences in gene expression between lesions and healthy endometrial tissues, highlighting the roles of inflammatory signaling, estrogen response, extracellular matrix remodeling, and immune cell infiltration [9–11]. However, while transcriptomic analyses have advanced our understanding of gene expression in endometriosis, they have largely overlooked the significance of RNA processing mechanisms such as alternative splicing, which can play a significant role in altering the transcriptome and proteome.

Alternative splicing is a key post-transcriptional regulatory mechanism that allows a single gene to generate multiple mRNA and protein isoforms by removing non-coding sequences (introns) and ligating only coding regions (exons) in different arrangements to produce a mature mRNA transcript. This process is orchestrated by the spliceosome, a dynamic ribonucleoprotein complex composed of five small nuclear RNAs (U1, U2, U4, U5, U6) and more than 200 associated proteins [12–14]. By selectively including or excluding specific exons, splicing enables cells to fine-tune gene function in a tissue-specific, developmental, or stimulus-dependent manner. Dysregulation of splicing can lead to the production of aberrant transcripts with altered stability, localization, or protein-coding capacity. Splicing errors have been implicated in numerous human diseases, including cancer, neurodegenerative disorders, and immune dysfunctions [15–19]. Moreover, emerging evidence suggests that splicing factors themselves are often mutated or misexpressed in disease contexts, further amplifying transcriptomic disruption [20]. In the context of endometriosis, the role of alternative splicing remains vastly underexplored, even though many processes relevant to lesion formation, such as cell proliferation, angiogenesis, and estrogen signaling, are regulated at the level of RNA splicing. Understanding how splicing is altered in endometriotic tissues may provide a critical missing link between observed transcriptional changes and disease pathology.

In this study, we investigate the landscape of alternative RNA splicing in endometriotic lesions by analyzing bulk RNA-seq data from endometriosis patients and healthy controls. Using replicate multivariate analysis of transcript splicing (rMATS), we identify widespread alterations in splicing patterns across both peritoneal and ovarian ectopic lesions. Compared to control endometrial tissue, endometriotic samples exhibit dysregulated exon inclusion, skipping, and mutually exclusive exon events. As proof of principle, we validate splicing alterations in several genes previously implicated in epithelial cell growth, metabolism, and immune regulation, including ZNF28 and GALNT7 and found alterations in splicing events. Our findings underscore the importance of RNA splicing as a regulatory layer contributing to the molecular complexity of endometriosis. By characterizing the splicing landscape of endometriotic lesions, this study expands our understanding of transcriptomic dysregulation and identifies alternative splicing as a potential contributor to endometrial cell growth, lesion development and disease heterogeneity.

## Results

**Fig. 1:**
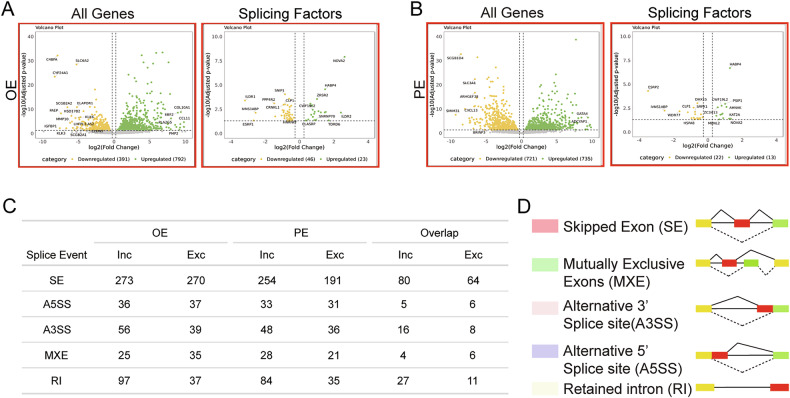
Altered mRNA splicing events in endometriosis. **A** Volcano plot of differentially expressed splicing factors in ovarian endometrium (OE). **B** Volcano plot of differentially expressed splicing factors in peritoneal endometrium. **C** Number of exclusion and inclusion splicing events relative to the five categories of AS evaluated by rMATS and caused by control (*n* = 2) vs Endometriosis ectopic patients (*n* = 3). The third column describes the size of the list of shared events across the two conditions. **D** Schematic representation of the total number of alternative splicing (AS) events identified in peritoneal (*n* = 3) and ovarian endometriotic lesions (*n* = 3) compared to control healthy endometrium (*n* = 3).

Previous studies have identified differentially expressed genes (DEGs) in endometriotic lesions compared to healthy endometrium. When we overlapped these DEGs (Data set: GEO GSE179640) from Control (*n* = 2), peritoneal endometriosis (PE (*n* = 3)), and ovarian endometriosis (OE (*n* = 3)), with known splicing factors, we discovered a substantial number of RNA-binding proteins (RBPs) among the differentially expressed genes. Specifically, in ovarian endometriotic lesions, we identified 46 downregulated and 23 upregulated RBPs, while peritoneal lesions showed 22 downregulated and 13 upregulated RBPs compared to the control endometrium, as shown in volcano plot analysis (Fig. 1A, B). Given this significant dysregulation of RBPs, we proceeded to investigate the broader alternative splicing landscape in endometriosis by analyzing bulk RNA-Seq data from patients with endometriotic lesions and healthy controls.

Our comprehensive analysis identified extensive alternative splicing alterations across five major splicing event categories: skipped exons (SE), alternative 5’ splice sites (A5SS), alternative 3’ splice sites (A3SS), mutually exclusive exons (MXE), and retained introns (RI) (Fig. 1D). Skipped exons were the most prevalent, with 254 inclusion and 191 exclusion events in ectopic peritoneal lesions, and 273 inclusion and 270 exclusion events in ectopic ovarian lesions (Fig. 1C). Among these, 80 inclusion and 64 exclusion events were shared between the two lesion types. Retained introns were also frequently observed, with 84 inclusion and 35 exclusion events in peritoneal lesions, and 97 inclusion and 37 exclusion events in ovarian lesions. Of these, 27 inclusion and 11 exclusion events overlapped between ectopic and ectopic ovarian lesions.

**Fig. 2:**
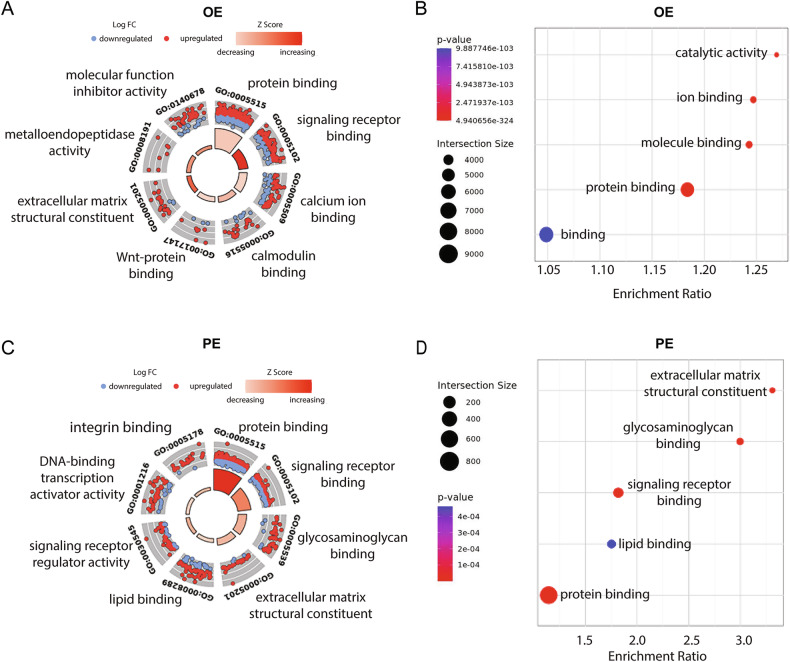
Integrative functional characterization of differentially expressed protein-coding genes. **A**, **C** The outer circle displays a scatter plot of the changes in expression levels for rhythmically expressed, clock-associated genes within each enriched Gene Ontology (GO) term. Red dots represent upregulated genes, while blue dots represent downregulated genes. The inner ring presents a bar plot, where bar height indicates the significance of GO term enrichment (−log₁₀ adjusted *P*-value). Bar color reflects the z-score: red indicates increased expression, orange indicates decreased expression. **B**, **D** Bubble plots display significantly enriched KEGG pathways for alternatively spliced genes in endometriosis patients. The size of each bubble represents the number of genes associated with the pathway, while the color indicates statistical significance (*P*-value). Panels (**A**, **B**) correspond to OE, and panels (**C**, **D**) correspond to PE.

To investigate the functional implications of the observed gene expression and splicing alterations, we performed Gene Ontology (GO) enrichment analysis and KEGG pathway analysis. Specifically, we analyzed differentially expressed protein-coding genes (adjusted *p* ≤ 0.05, fold change ≥20%) and alternatively spliced genes (FDR ≤ 0.05, inclusion level difference ≥20%) for enrichment in molecular function GO terms and KEGG pathways. Analysis of the 735 upregulated and 721 downregulated genes from ovarian endometriotic lesions revealed significant enrichment for KEGG and GO pathways such as protein binding, signaling receptor binding, glycosaminoglycan binding, extracellular matrix structural constituent, and lipid binding (Fig. 2A). Enrichment of similar pathways was also observed among alternatively spliced genes (Fig. 2B). The 792 upregulated and 391 downregulated genes from peritoneal endometriotic lesions were significantly enriched for KEGG and GO pathways related to protein binding and ion binding (Fig. 2C). Similarly, alternatively spliced genes showed enrichment for related pathways (Fig. 2D). DEGs from both ovarian and peritoneal endometriotic tissue were enriched for GO terms such as protein binding, signaling receptor binding, and extracellular matrix structural constituent. However, the balance of up- and down- regulated genes, represented by the z-score, had slight differences between the disease tissue types. The protein binding term (GO:0005515) was the most significant enriched pathway for both tissues. This pathway was increased in both ovarian and peritoneal endometriotic lesions (Fig. 2A, B), but its increase in ovarian lesions was more pronounced than in the peritoneal lesions. The signaling receptor binding term (GO:0005102) was also increased in both lesion types, however peritoneal endometriotic lesions had a more pronounced increase than the ovarian lesions (Fig. 2A, B). The extracellular matrix structural constituent term (GO:0005201) was also increased in both lesion types and peritoneal endometriotic lesions had a slightly higher increase than the ovarian lesions. Besides having variable impact on overlapping GO pathways, DEGs from ovarian and peritoneal lesion also were enriched in distinct GO term pathways (e.g. integrin binding and metalloendopeptidase activity) (Fig. 2A, B), suggesting tissue-specific gene expression alterations for diseased tissue.

**Fig. 3:**
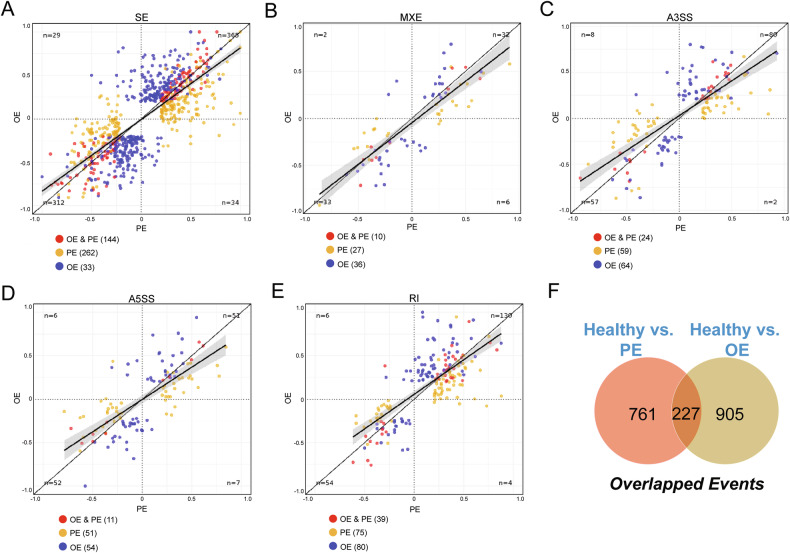
Common and distinct AS events across lesion types. Scatterplots show relative splicing abundance for each tool (rMATS–InclusionLevelDifference) and each event type (SE spliced exon, RI retained intron, MXE Mutually exclusive exons, A5SS alternative 5’ splice site, A3SS alternative 3’ splice site). The linear regression line is shown in black. **A** SE events; **B** MXE events; **C** A3SS events; **D** A3SS events; **E** RI events; **F** Venn diagram showing the number of overlapping and unique AS events between peritoneal and ovarian lesions.

To evaluate the robustness of our splicing analysis, we examined the overlap among different splicing event types. Scatterplots demonstrated strong correlations across all splicing event categories (Fig. 3A–E). Comparative analysis of peritoneal and ovarian lesions revealed both shared and unique splicing events. Specifically, Venn diagram analysis identified 227 overlapping alternative splicing events between the two lesion types, with 761 events unique to peritoneal lesions and 905 unique to ovarian lesions (Fig. 3F). These results indicate that although endometriotic lesions share common splicing alterations, each anatomical location also exhibits distinct molecular signatures, potentially contributing to disease heterogeneity.

### Proof of principle validation for the AS event in candidate genes, *ZNF28* and *GALNT7*

**Fig. 4:**
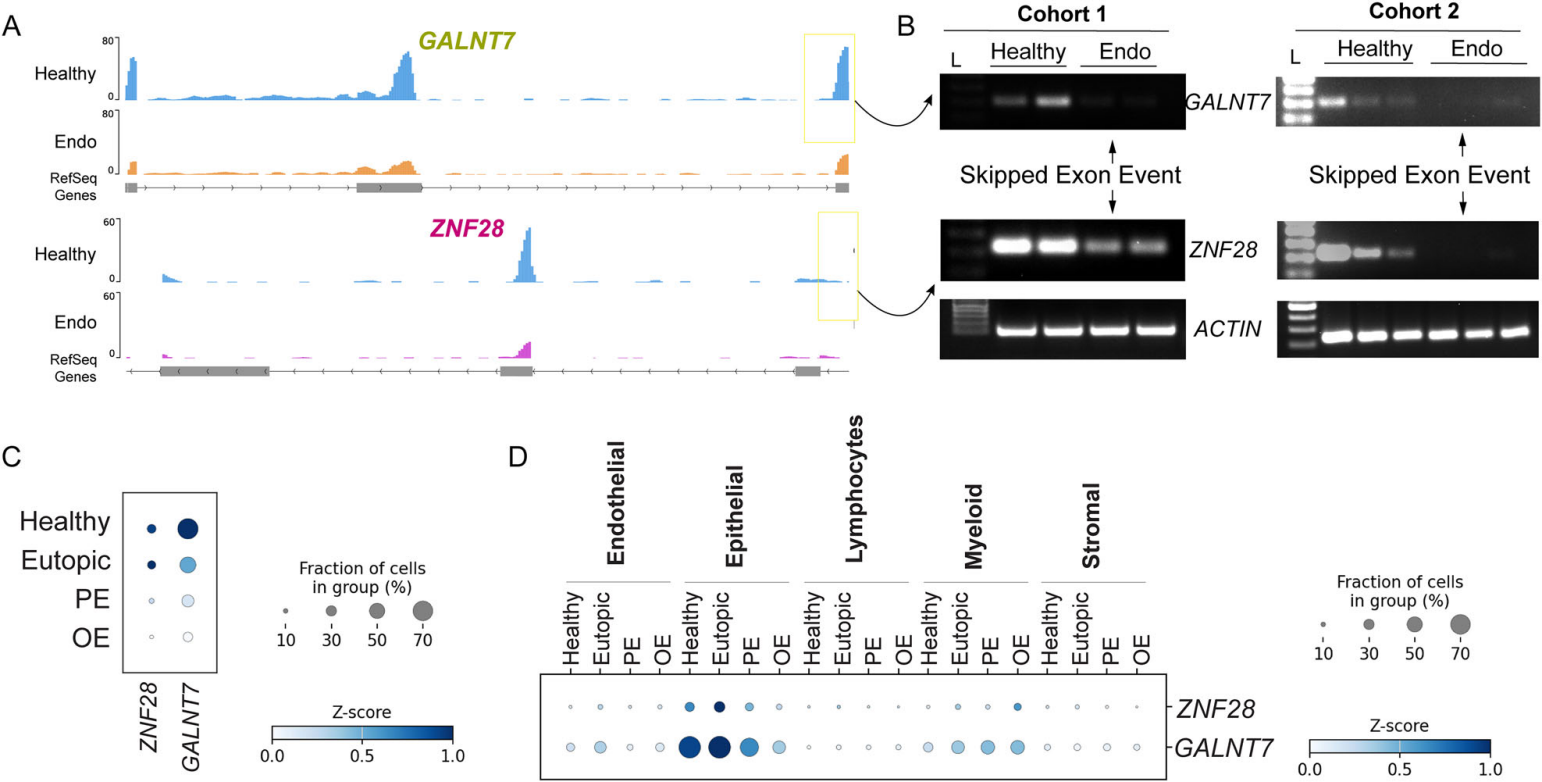
Validation of altered mRNA splicing events in *ZNF28* and *GALNT7* genes. **A** Sashimi plots generated with the IGV genome browser illustrating skipped exon (SE) events in two candidate genes, *ZNF28* and *GALNT7*, in lesions compared to controls. **B** RT-PCR validation of SE events using primers flanking the excluded exons of *ZNF28* and *GALNT7*. cDNA for cohort 1 was generated from healthy endometrium and peritoneal lesion tissues (*n* = 2), and cohort 2 was generated from healthy endometrium and peritoneal lesion tissues (*n* = 3) obtained from Dr. Tayade. **C** Expression levels of GALNT7 and ZNF28 in Healthy and Endometriotic patients. **D** GALNT7 and ZNF28 expression levels in different cell subtypes.

Given the widespread alterations in alternative splicing events observed in endometriotic lesions, we next sought to identify specific splicing events in specific genes and validate as proof of principle. The genes were prioritized based on a false discovery rate (FDR) threshold of <0.05 and a minimum inclusion level difference (ILD) of ≥0.2 or ≤−0.2, indicating significant exon inclusion or exclusion, respectively. From our dataset, we selected two candidate genes, *ZNF28* and *GALNT7*, which exhibited consistent reduced exon inclusion events across both ovarian and peritoneal lesion types (Fig. 4A). To validate this AS event, specific primers were designed to flank the alternatively spliced regions, specifically exon 3 in *ZNF28* and exon 6 in *GALNT7*. RT-PCR was performed using cDNA derived from healthy endometrial tissue and peritoneal lesion samples. Consistent with our splicing analysis, RT-PCR validation confirmed the truncation of exon 3 in *ZNF28* and exon 6 in *GALNT7* specifically in endometriotic lesions, highlighting their possible role in disease-associated transcriptomic reprogramming (Fig. 4B). To further validate our findings, we utilized the single-cell RNA-seq dataset kindly provided by Dr. Ellis Courtois [10]. In this dataset, scRNA-seq was performed on biopsies from 14 individuals. Control eutopic endometrium (Ctrl) samples were obtained from non-endometriosis patients, while eutopic endometrium (EuE), ectopic peritoneal lesions (PE) with their adjacent regions (EcPA), and ectopic ovarian lesions (OE) were collected from revised ASRM Stage II–IV patients, most of whom were under similar hormonal treatment. In total, this database generated 108,497 single-cell transcriptomes, with a median of 9186 unique transcripts and 2823 genes per cell. Cells were classified into five primary types: epithelial, stromal, endothelial, lymphocyte, and myeloid. In line with our above results, we observed that both ZNF28 and GALNT7 were expressed at higher levels in control non-endometriosis patients compared to peritoneal and ovarian ectopic lesions (Fig. 4C). Interestingly, we observed that epithelial cells from control and eutopic endometrium showed higher levels of these genes compared to other cell types, while their expression was significantly downregulated in peritoneal and ovarian lesions (Fig. 4D). Collectively, these results suggest that both ZNF28 and GALNT7 are highly expressed in epithelial cells of the normal endometrium and are downregulated in endometrial lesions, potentially due to exon-skipping splicing alterations.

### GALNT7 and ZNF28 inhibit the endometriotic cell growth, a potential protective role in endometriosis

**Fig. 5:**
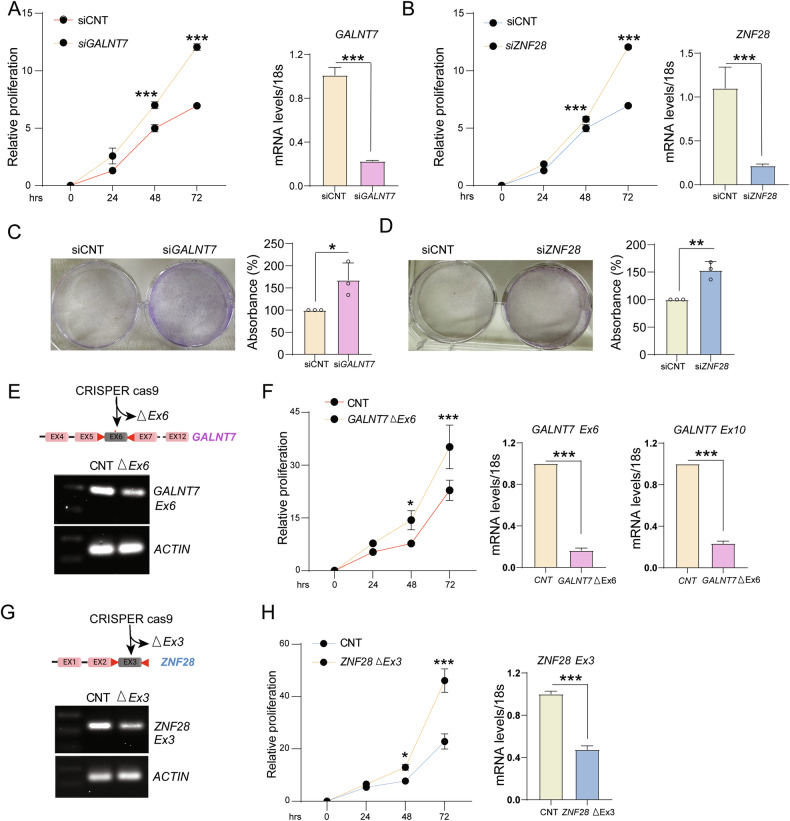
Knockdown of *GALNT7* and *ZNF28* increased the proliferation of IHEEC cells. **A**, **B** Cell viability was assessed by MTT assay at multiple time points post-transfection. Absorbance was measured at 570 nm. *GALNT7 and ZNF28* transcript levels were measured by QPCR. Representative clonogenic assays (left) and quantitation (right) in *GALNT7* (**C**) and *ZNF28* (**D**) knockdown IHEEC cells. **E** RT-PCR validation of *GALNT7* using primers flanking the excluded exon 6. cDNA for RT-PCR was generated from CRISPR Cas9 Exon 6 deleted IHEEC cells. **F** Cell viability was assessed by MTT assay at multiple time points post-transfection. Absorbance was measured at 570 nm. *GALNT7* Exon 6 *and* Exon 10 transcript levels were measured by QPCR. **G** RT-PCR validation of *ZNF28* using primers flanking the excluded exon 6. cDNA for RT-PCR was generated from CRISPR Cas9 Exon 3 deleted IHEEC cells. **H** Cell viability was assessed by MTT assay at multiple time points post-transfection. Absorbance was measured at 570 nm. *ZNF28* Exon 3 transcript levels were measured by QPCR. Data are presented as the mean ± SEM from a representative experiment. Statistical significance is indicated as *p* < 0.01 (**) and *p* < 0.001 (***). Results are representative of three independent experiments.

Given the observed reduction in exon events of GALNT7 and ZNF28 in endometrial lesion tissues, we hypothesized that they may play a functional role in regulating the proliferation of endometrial cells. To test this, we selected Human endometrial epithelial cells (IHEEC) and performed transient knockdown using siRNA specifically targeting GALNT7 and ZNF28. Cells transfected with a non-targeting control siRNA served as negative controls. To confirm the efficiency of SF3B1 silencing, we performed quantitative real-time PCR (qRT-PCR) analyses. As shown in Fig. 5A, B, GALNT7 and ZNF28-targeted siRNA led to a marked reduction in their mRNA levels in IHEEC cells, confirming effective knockdown. Following siRNA transfection, cells were replated after 48 h and monitored for cell proliferation over 72 h. As shown in Fig. 5A, B, GALNT7 and ZNF28 knockdown resulted in a significant increase in cell proliferation of IHEEC cells compared to control-treated cells. To further validate the impact of their depletion on the long-term proliferative capacity of endometrial epithelial cells, we performed clonogenic assays. Notably, GALNT7 silencing increased colony formation 50%, whereas ZNF28 knockdown showed an upsurge by 70%, as shown in Fig. 5C, D. To assess the functional importance of GALNT7 and ZNF28 splice variants, we generated IHEEC cells with efficient deletion of exon 6 for GALNT7 and exon 3 for ZNF28 using CRISPR-Cas9 technology (Fig. 5E, G). We found that efficient deletion of exon 6 in GALNT7 and exon 3 in ZNF28 resulted in a significant reduction in overall transcript levels, likely due to skipped exon-induced transcript instability and subsequent mRNA degradation, implying that these variants are essential for maintaining transcript integrity. Importantly, exon-specific deletion in both these two genes led to a significant increase in cell proliferation (Revised Fig. 5F, H), phenocopying the effects observed following whole-transcript knockdown of *GALNT7* and *ZNF28* (Fig. 5A, B). These data show that GALNT7 and ZNF28 regulate the proliferative capacity of endometrial epithelial cells, and their loss due to aberrant splicing markedly increases both short‑term growth and long‑term clonogenic potential. Together, our results support a role for altered alternative mRNA splicing in endometriosis.

## Discussion

Endometriosis is a prevalent, chronic, and hormone-dependent benign gynecological disorder marked by the presence of endometrial glands and mesenchymal stromal cells outside the uterine cavity. Affecting ~10% of women of reproductive age globally, endometriosis imposes significant physical, emotional, and economic burdens. Despite substantial advances, our understanding of the underlying molecular mechanisms driving the development and progression of endometriosis remains poorly defined. In this study, we conducted a comprehensive analysis of precise and quantitative bulk RNA-Seq data freely available online (GEO GSE179640) to identify alternative splicing events that characterize the splicing landscape of endometriosis patients. We identified 761 alternative splicing (AS) events in peritoneal lesions and 905 AS events in ovarian lesions, primarily impacting protein-coding genes. These events occurred at a higher frequency compared to control eutopic endometrium. These findings support a widespread role for AS events in generating proteomic diversity and endometriosis progression. Notably, our RT-PCR analysis confirmed that the exon inclusion of two genes, ZNF28 and GALNT7, was significantly reduced in lesions. Given that our GO analysis enabled a precise comparison of AS events in ZNF28 and GALNT7, we were able to reveal functional consequences associated with proliferation pathways. Functional assays demonstrated that truncation of ZNF28 and GALNT7 promotes the proliferation of endometrial epithelial cells, suggesting a link between aberrant splicing and lesion development in endometriosis.

Recent transcriptomic studies, including both bulk and single-cell RNA sequencing, have revealed widespread gene expression changes in ectopic lesions compared to healthy eutopic endometrium, highlighting alterations in immune signaling, hormone responsiveness, and cell–cell communication pathways. These studies have uncovered lesion- and cell-type-specific transcriptional signatures in endometriosis, including dysregulation of inflammatory mediators, growth factors, and estrogen-regulated genes. However, a majority of these studies have focused on gene-level expression changes, often overlooking the complexity introduced by pre-mRNA splicing. This major gap limits our understanding of endometriosis pathophysiology, especially given that most human genes produce multiple mRNA isoforms and that splicing outcomes dramatically influencing protein function, stability, and localization. Emerging evidence in other estrogen-driven diseases, such as breast cancer, suggest that hormone signaling can influence not just transcription, but also splicing that generates disease-specific mRNA variants [21]. Given the central role of estrogen in endometriosis, it is plausible that similar hormone-driven splicing changes generate unique, functionally relevant mRNA isoforms in endometriotic cells. Yet, a comprehensive analysis of splicing alterations and the resulting mRNA variants in endometriosis remains to be fully elucidated.

Aberrant splicing events occur when the cellular machinery incorrectly removes or joins segments of pre-mRNA. Such splicing errors can result in premature transcription termination, abnormal splicing events (such as exon mis-inclusion, intron retention, or cryptic splice site usage), or defective mRNA processing, particularly due to aberrant polyadenylation [22–25]. These disruptions produce mRNA variants that disrupt gene expression and protein function and can contribute to aberrant mRNA variants in various diseases, including cancer, neurodegenerative disorders, inherited genetic conditions [26–28] and amyotrophic lateral sclerosis (ALS) [29–31]. In our comprehensive analysis, we identified dozens of differentially expressed splicing factors and hundreds of alternative splicing (AS) events across five major event types in both ovarian and peritoneal lesions. Skipped exons and retained introns were the most prevalent. GO enrichment analysis revealed that genes affected by splicing changes were enriched in pathways involved in protein, lipid, and calcium ion binding, indicating that splicing alterations may have broad biological consequences in endometriosis pathogenesis. Notably, comparative analysis revealed both shared and peritoneal and ovarian lesion-specific splicing events, with 227 common events and over 1600 events unique to either peritoneal or ovarian lesions, reflecting the underlying molecular heterogeneity of this disease.

Among the genes we identified with altered splicing, we focused on two candidate genes, GALNT7 and ZNF28, that showed altered exon inclusion. Specifically, GALNT7 (exon 6) and ZNF28 (exon 3 and 4) exhibited decreased inclusion in ectopic tissues compared to healthy controls. *GALNT7* encodes a key enzyme in the glycosylation pathway, responsible for transferring N-acetylgalactosamine (GalNAc) to serine or threonine residues on target proteins, an essential step in initiating O-linked glycan biosynthesis [32]. *ZNF28*, a member of the zinc finger protein family, functions primarily as a transcription factor that binds DNA to regulate gene expression; however, detailed characterization of this protein remains. In addition, both *GALNT7* and *ZNF28* have been implicated in oncogenic processes, with roles in critical biological functions including cell cycle progression, metabolism, immune regulation, and tumorigenesis. Interestingly, our analysis of endometriotic lesions revealed that exons in both genes were preferentially truncated, likely resulting in reduced production of mature mRNA. Consistent with this, expression levels of *GALNT7* and *ZNF28* were significantly lower in endometriosis patients lesions compared to control endometrium. Importantly, our analysis revealed that epithelial cells expressed higher levels of ZNF28 and GALNT7 compared to other cell types, whose expression is being reduced in patients with endometriosis. In endometriosis, abnormal epithelial cells contribute to the development of lesions and promote their invasiveness. These findings suggest that inhibition of ZNF28 and GALNT7 may enhance the proliferative potential of epithelial cells, thereby driving disease progression. In line with these findings, we observed that suppressing GALNT7 and ZNF28 led to an increased proliferation rate of endometrial epithelial cells, suggesting a potential role in regulating cell growth. This suggests a potential growth-suppressive role in the context of endometriosis, which contrasts with their more commonly reported oncogenic activity in cancers. Remarkably, we found that, unlike epithelial cells, myeloid cells displayed relatively higher expression of ZNF28 and GALNT7 in endometriotic lesions. Previous studies have demonstrated that GALNT7 plays a critical role in regulating the inflammatory phenotype of macrophages in cancers [33]. Based on these observations, our results suggest that ZNF28 and GALNT7 in myeloid cells help regulate chronic inflammation, a key factor in driving the progression of endometriosis. Notably, these findings indicate that alternative splicing may occur in a cell-type–specific manner, contributing to disease development and progression. To better define their roles in endometriosis pathophysiology, large-scale studies in diverse patient cohorts are needed to identify functional genetic variants, elucidate the regulatory mechanisms underlying their altered splicing and expression, and clarify how alternative splicing modulates cellular behavior in endometriosis.

Genome-wide association studies (GWAS) have also uncovered several genetic variants associated with endometriosis risk, particularly SNPs near genes involved in inflammation, hormone signaling, and cell adhesion, biological processes central to disease pathogenesis [34, 35]. However, GWAS typically identify associations between SNPs and disease risk without revealing the underlying functional mechanisms. Increasingly, splicing quantitative trait loci (sQTLs), SNPs that influence splicing patterns, are being recognized as critical drivers of disease [36, 37]. Integrating GWAS-identified SNPs with alternative splicing data from RNA sequencing can help pinpoint functional variants that may drive disease through splicing regulation. However, analyzing splicing data from a single patient provides a limited view, as it only captures individual-specific AS-SNP associations. This narrow approach may fail to detect sQTLs relevant across the population and miss the full range of functionally significant variants. Therefore, integrative studies using large, genetically diverse cohorts of endometriosis patients are essential in capturing an accurate understanding of splicing associated variants in endometriosis. Ultimately, these efforts will uncover novel disease mechanisms, enhance our understanding of the genetic regulation of splicing in endometriosis and inform the development of diagnostic and therapeutic strategies targeting mRNA processing.

## Materials and methods

### RNA sequencing data processing

Bulk RNA sequencing datasets were obtained from the Gene Expression Omnibus database (GEO accession: GSE179640). In total, 12 eutopic, 6 ovarian lesions, and 6 peritoneal lesions from bulk RNA-seq samples were retrieved from the database and processed for comprehensive RNA sequencing analysis. Raw sequencing data underwent a comprehensive quality assessment using FastQC v0.11.9, which evaluated read quality metrics and adapter content. Subsequently, read preprocessing and adapter removal were performed using fastp v0.20.0. High-quality processed reads were aligned to the human reference genome using STAR v2.7.9a. The STAR alignment index was constructed using FASTA sequences and GTF annotation files obtained from the GENCODE portal (human genome assembly GRCh38, release v28). Quality control metrics for both sequencing reads and genome alignments were systematically compiled using MultiQC v1.12. Resulting alignment files were indexed using SAMtools. Genome-wide coverage visualization tracks were generated using bamCoverage from the deepTools suite, employing counts per million (CPM) normalization with 5 bp resolution binning. Following the comprehensive analyses as described above, only 2 control samples, 3 peritoneal endometriosis samples, and 3 ovarian endometriosis samples met the quality criteria for splicing analysis and were included in this study.

### Identification of differentially expressed genes

Raw gene-level read counts were quantified through STAR alignment using the --quantMode GeneCountsparameter. Differential gene expression analysis was performed using DESeq2, following count data normalization. To ensure robust statistical analysis, genes with mean read counts below 10 across all samples were excluded from downstream analysis. Significantly differentially expressed genes were identified using stringent criteria: an adjusted *p*-value (FDR) threshold of 0.05 combined with a log₂ fold change threshold of ±0.263, corresponding to a minimum 20% expression change. Differential expression results were visualized using R v4.1.2 with the ggplot2 and pheatmap packages.

### Analysis of alternative splicing events

Alternative splicing patterns were comprehensively analyzed using rMATS v4.1.2 with STAR-generated BAM alignment files and GENCODE v33 gene annotations for the human reference genome GRCh38. The analysis encompassed five distinct alternative splicing event categories: exon skipping, intron retention, mutually exclusive exons, alternative 5′ splice sites, and alternative 3′ splice sites. Statistical significance was determined using an FDR threshold of 0.05, while biological relevance was assessed based on inclusion level differences (ILD) of ≥0.2 or ≤−0.2, representing substantial alterations in exon inclusion versus exclusion patterns. Selected splicing events underwent manual validation through detailed visualization in the Integrative Genomics Viewer (IGV) using sample-specific bigWig coverage tracks. Sashimi plots and comprehensive genome coverage visualizations were generated using pyGenomeTracks to provide high-resolution views of splicing junction patterns and splice site usage.

### Functional enrichment analysis

Comprehensive gene set enrichment analysis was performed on significantly differentially expressed genes using gProfiler. Functional categories were considered significantly enriched at an adjusted *p*-value threshold of 0.05. Gene Ontology (GO) term visualization was accomplished using the GO bubble plot function from the gprofiler2 R package and the GOCircle plot from the GOplot R package. All statistical analyses and visualizations were conducted in R version 4.4.2.

### Cell culture

Immortalized Human Endometriotic Epithelial Cells expressing Luciferase (iHEECs/Luc) (generously provided by Dr. Sang Jun Han from Baylor College of Medicine) were cultured in Dulbecco’s Modified Eagle Medium/Nutrient Mixture F-12 (DMEM/F12, Gibco: 11320-033) with 10% fetal bovine serum (FBS, Gibco: 26140-079) and 1% penicillin-streptomycin (Gibco: 15140-122), and 2.5 mg/ml amphotericin-B to support optimal growth conditions [38]. Cells were maintained at 37 °C in a humidified incubator with 5% CO₂. Cells were passaged at 80% confluency using 0.01% trypsin-EDTA (Gibco: 25300-054) and fresh media was added the following day.

### siRNA transfections

IHEEC cells were seeded in 6-well plates at a density of 1.5 × 10⁵ cells per well and allowed to adhere overnight. Cells were then co-transfected with 60 pmol of either GALNT7- or ZNF28-specific siRNA, or a non-targeting control siRNA (ON-TARGETplus SMARTpool siRNA; Horizon Discovery), using Lipofectamine RNAiMAX Transfection Reagent (Invitrogen: 13778030) in Opti-MEM Reduced Serum Medium (Gibco, Cat# 31985-070), following the manufacturer’s protocol. After a 6-h incubation with the transfection complex, the medium was replaced with complete growth medium (DMEM/F12; Gibco: 11320-033). Transfected cells were harvested after 36 h and reseeded into 96-well plates for MTT assays or 6-well plates for clonogenic assays. All experiments were independently repeated at least three times to ensure reproducibility.

### CRISPR-Cas9 transfection

IHEEC cells were seeded in a 12-well plate at a density of 8 × 10^4^ cells per well and allowed to adhere overnight. Cells were then co-transfected with 2 µM of either GALNT7 (Exon 6) or ZNF28 (Exon 3) or a non-targeting control guide RNA oligos, along with 1 µM Cas9 (Alt-RTM CRISPR-Cas9; IDT) using Lipofectamine™ CRISPRMAX™ Cas9 Transfection Reagent (Invitrogen: CMAX00001) in Opti-MEM Reduced Serum Medium (Gibco, Cat# 31985-070), following the manufacturer’s protocol. Transfected cells were harvested after 48 h and reseeded into 96-well plates for MTT assays. All experiments were independently repeated at least three times to ensure reproducibility.

### MTT assay

MTT assays were performed using the *CellTiter 96® Non-Radioactive Cell Proliferation Assay* (Promega: G4000) according to the manufacturer’s instructions. Briefly, IHEEC cells were transfected as described above. Cells were counted using a hemocytometer and seeded at a density of 5 × 10³ cells per well in 96-well plates. Cell proliferation was assessed at 0, 24, 48, and 72 h after seeding. Absorbance was measured using a microplate reader (BioTek). Proliferation rates were calculated relative to the 0-h time point.

### Clonogenic assay

Post-transfection, IHEEC cells were seeded into 6-well plates at a density of 5 × 10³ cells per well and maintained for 14 days to allow colony formation. Media was changed every 1–2 days. After 14 days, colonies were then fixed with 4% paraformaldehyde (Thermofisher scientific: 28908) for 10 min at room temperature and stained with 0.1% crystal violet solution (Need catalogue number) for 15 min. Colonies were counted manually using ImageJ software. For quantitative analysis, the retained crystal violet stain was removed using 10% acetic acid, and eluted stain absorbance was measured at 490 nm using a spectrophotometer (Infinite M1000).

### RT-PCR for validation

To validate exon exclusion AS events, 1000 ng of RNA was reverse-transcribed using the High-Capacity cDNA Reverse Transcription Kit (Thermo Scientific, Waltham, MA, USA). The amplified cDNA was diluted to a concentration of 10 ng/μL and subjected to PCR using sequence-specific primers listed in Supplementary Table S1 (see Supplemental Information for primer sequences and annealing temperatures). Briefly, PCR was carried out for 30 cycles under the following conditions: initial denaturation at 95 °C for 5 min; followed by 30 s at 95 °C for denaturation, 30 s at 50 °C for annealing, and 45 s at 72 °C for extension; with a final extension at 72 °C. PCR products were analyzed by agarose gel electrophoresis, and images were captured using a Bio-Rad imaging system.

### RNA isolation and real-time polymerase chain reaction (qPCR)

Total RNA was isolated from IHEEC cells using the RNeasy Total RNA Isolation Kit (Invitrogen: 12183018), in accordance with the manufacturer’s instructions. RNA quality and concentration were assessed using a NanoDrop spectrophotometer (Nanodrop ONE Thermoscientific). One microgram of the isolated RNA was reverse transcribed into complementary DNA (cDNA) using the TaqMan Reverse Transcription Kit (Applied Biosystems: Need number), following the manufacturer’s protocol. Quantitative real-time PCR was conducted using the ABI Prism 7700 Sequence Detection System with TaqMan 2X Master Mix and validated primers (Applied Biosystems, USA) and normalized to the expression of 18S ribosomal RNA as an internal control. Amplification reactions were run in 20 µL volumes using TaqMan Universal PCR Master Mix on the ABI Prism 7700 Sequence Detection System. Relative gene expression levels were calculated using the ΔΔCt method.

### Human subjects tissue collection

Cohort 1 tissues were collected at Washington University in St. Louis following written informed consent under an Institutional Review Board-approved protocol (IRB ID: 201612127). Cohort 2 tissues were kindly provided by Dr. Chandra Tayade and were collected at Kingston Health Sciences Center, Queen’s University (Kingston, ON, Canada). Ectopic lesions were obtained from women with endometriosis undergoing excision surgery. Control endometrial biopsies were collected from healthy women during the proliferative phase of the menstrual cycle or from women undergoing elective tubal ligation.

## Supplementary information

Supplementary Table S1

## Data Availability

siRNA and cell lines used in this study will be made available upon request, but we may require payment and/or a completed materials transfer agreement if there is potential for commercial application.

## Code availability

Bulk RNA sequencing datasets were obtained from the Gene Expression Omnibus database (GEO accession: GSE179640) [10].
