# Correction: Misregulated alternative splicing in endometriosis: a role for aberrant mRNA variants in endometriotic cell growth

**DOI:** 10.1038/s41420-026-03154-3

**Published:** 2026-05-29

**Authors:** Venkata Naga Goutham Davuluri, Michelle Dias, Roxanna Llinas, Neha Kamath, Pooja Popli, Shannon M. Hawkins, Chandrakant Tayade, Elise T. Courtois, Hari Krishna Yalamanchili, Ramakrishna Kommagani

**Affiliations:** 1 Department of Pathology and Immunology, Baylor College of Medicine, Houston, TX USA; 2 Department of Pediatrics, Baylor College of Medicine, Houston, TX USA; 3 Jan and Dan Duncan Neurological Research Institute, Texas Children’s Hospital, Houston, TX 77030, USA; USDA/ARS Children’s Nutrition Research Center, Department of Pediatrics, Baylor College of Medicine, Houston, TX USA; 4 Department of Obstetrics and Gynecology, Indiana University School of Medicine, Indianapolis, IN USA; 5 Department of Biomedical and Molecular Sciences, Queen’s University, Kingston, ON Canada; 6 The Jackson Laboratory for Genomic Medicine, Farmington, CT USA; 7 UConn Health School of Medicine, OB/Gyn Department, Farmington, CT USA; 8 USDA/ARS Children’s Nutrition Research Center, Department of Pediatrics, Baylor College of Medicine, Houston, TX USA; 9 Department of Molecular Virology and Microbiology, Baylor College of Medicine, Houston, TX USA; 10 Center for Drug Discovery, Baylor College of Medicine, Houston, USA

**Keywords:** Alternative splicing, Endocrine reproductive disorders

Correction to: *Cell Death Discovery* 10.1038/s41420-026-03015-z, published online 15 March 2026

In this article the author’s name ‘Neha Kamath’ has been published wrongly as ‘Kamath Neha’.

The original article has been corrected.

