# Supplementary material for: Misregulated alternative splicing in endometriosis: a role for aberrant mRNA variants in endometriotic cell growth
Source: Cell Death Discov. 2026 Mar 15;12:149. doi: 10.1038/s41420-026-03015-z (PMC13039441; doi:10.1038/s41420-026-03015-z)
Supplement: Supplementary file 1 — Supplementary Table S1 [file 41420_2026_3015_MOESM1_ESM.docx]

**Table S1. List of primers and TaqMan probes**

| **Gene name** | **Species** | **Application, Chemistry** | **Company** | **Sequence/Cat. No.** |
| --- | --- | --- | --- | --- |
| *GALNT7* | Human | qPCR, Taqman | ABI | [Hs00213624_m1](https://www.thermofisher.com/taqman-gene-expression/product/Hs00213624_m1?CID=&ICID=&subtype=) |
| *ZNF28* | Human | qPCR, Taqman | ABI | Hs00867578_s1 |
| GALNT7 Exn-6 Forward | Human | PCR | Sigma | CCGCTTATAGATGTCATA |
| GALNT7 Exn-6 Reverse | Human | PCR | Sigma | TTCTCAGTCTCTTCTCTT |
| ZNF28 Exn-3 Forward | Human | PCR | Sigma | TATAATTCTCCAGCATCAC |
| ZNF28 Exn-3 Reverse | Human | PCR | Sigma | GGTCTATTGACATTCAGG |
